# Data set for transcriptome analysis of *Escherichia coli* exposed to nickel

**DOI:** 10.1016/j.dib.2016.08.069

**Published:** 2016-09-07

**Authors:** Manon Gault, Agnès Rodrigue

**Affiliations:** Microbiologie, Adaptation et Pathogénie, UMR5240, INSA Lyon, Université Lyon 1, CNRS, Université de Lyon, F-69621 Villeurbanne, France

**Keywords:** RNA-Seq, *Escherichia coli*, nickel

## Abstract

Ni is recognized as an element that is toxic to humans, acting as an allergen and a carcinogenic agent, and it is also toxic to plants. The toxicity of Ni has been understudied in microorganisms. The data presented here were obtained by submitting the model bacterium *Escherichia coli* K-12 to nickel stress. To identify expressed genes, RNA-Seq was performed. Bacteria were exposed to 50 µM NiCl_2_ during 10 min. Exposure to Ni lead to the deregulation of 57% of the *E. coli* transcripts. Further analysis using DAVID identified most affected biological pathways. The list of differentially expressed genes and physiological consequences of Ni stress are described in “Ni exposure impacts the pool of free Fe and modifies DNA supercoiling via metal-induced oxidative stress in *Escherichia coli* K-12” (M. Gault, G. Effantin, A. Rodrigue, 2016) [1].

**Specifications Table**

**Table:** 

Subject area	*Biology*
More specific subject area	*Bioinformatics and microbiology*
Type of data	*Tables, figure*
How data was acquired	*High-throughput RNA-sequencing*
Data format	*Filtered and analyzed with statistical tests*
Experimental factors	*The bacteria were grown in minimal medium until early log-phase where 50* *µM NiCl*_*2*_*was added, after 10* *min bacteria were harvested and frozen*
Experimental features	*Total RNA was extracted using the frozen acid-phenol method. ARNr were excluded. Directional libraries were sequenced on Illumina Hiseq2500 in single reads.*
Data source location	*Laboratory “Microbiologie, Adaptation et Pathogénie”, UMR5240, INSA Lyon, France*
Data accessibility	*Data are with this article and deposited in NCBI׳s Gene Expression Omnibus (GEO), accessible through GEO Series accession number GEO:*GSE76167http://www.ncbi.nlm.nih.gov/geo/query/acc.cgi?acc=GSE76167

**Value of the data**

• Ni, as many transition metals, is essential as a trace element to living organisms whereas it becomes toxic when present in excess. At present, the description of Ni toxicity in bacteria is under-studied although this metal is a widespread element bacteria are in contact with.
• The data shows differentially expressed genes under Ni stress that could be compared to differentially expressed genes in other metal-stress conditions or other stress conditions.
• Analysis of the biological pathways impacted when cells are exposed to Ni will help to understand the molecular mechanisms of Ni- or metal-stress.
• Identification of Ni-deregulated genes could lead to biotechnological applications such as the design of whole cell biosensors.

## 1 Data

**Fig. 1:**
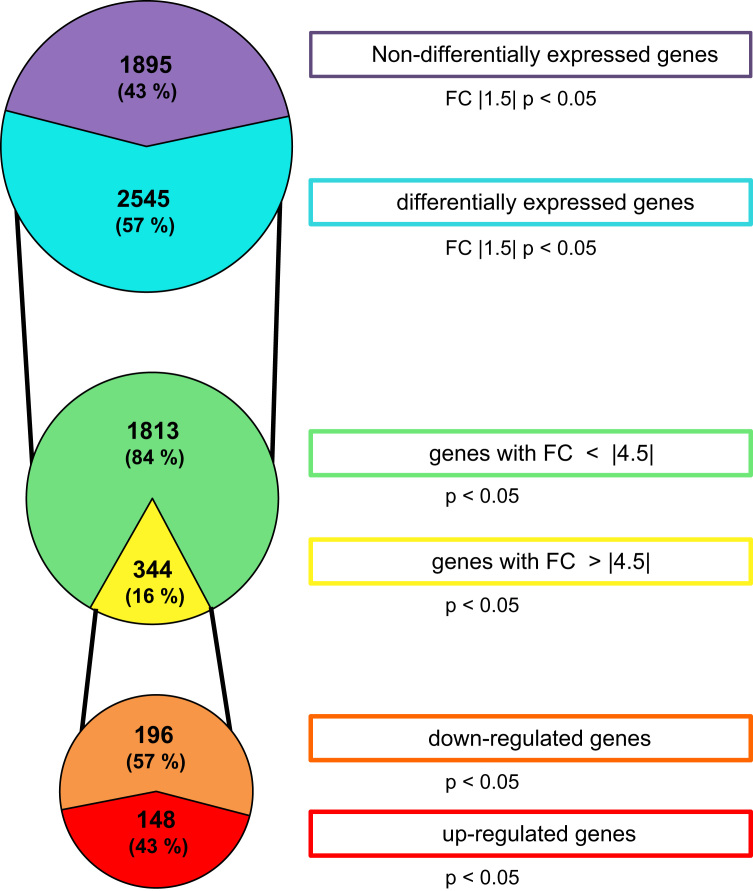
*E. coli* transcriptome response to Ni. The diagrams show the number of genes whose synthesis was increased or decreased after metal exposure. 57 % of the 4440 *E. coli* transcripts were differentially expressed using expression ratio of 1.5 (*p*<0.05). Among these 57 %, 16 % had expression ratios superior to 4.5. The functional analysis was performed on these 16% that represent 196 down-regulated and 148 up-regulated genes.

The RNA-Seq and gene expression datasets were deposited in NCBI׳s Gene expression Omnibus [2], accessible through GEO series accession number GEO: GSE76176. Fig. 1 shows the distribution of deregulated genes in *E. coli* upon exposure to 50 µM Ni. 2545 genes were deregulated considering a Fold-Change (FC) of 1.5, representing 57 % of the 4440 annotated transcripts of *E. coli* K-12 strain W3110. Gene Ontology was applied to classify differentially expressed genes according to their biological function (see Fig. 1 in [1]). GO Terms that were enriched in the list of differentially expressed genes were identified using the DAVID tools (Database for Annotation, Visualization and Integrated Discovery) [3], [4]. Pathways that were significantly affected were mapped using KEGG and are listed in Table 1.

## 2 Experimental design, materials and methods

### 2.1 Strains and growth conditions

*E. coli* K-12 cells were grown at 37 °C in minimal medium supplemented with glucose until O.D._600 nm_= 0.3 and then treated with 50 µM NiCl_2_ during 10 min. These conditions lead to maximal expression of the Ni-stress marker gene *rcnA* (see Fig. S1A and S1B in [1]).

### 2.2 RNA extraction and RNA-Seq

Three samples of each condition were treated, as described in [1].

### 2.3 Data analysis

Strand-orientated RNA-Seq was performed on Illumina Hiseq2500. Basecalls were performed using HCS 2.0.5 and RTA 1.17.20. Reads were aligned to whole reference genome *Escherichia coli* K-12 W3110 NC_007779 using CASAVA v 1.8.2 software (Illumina). Gene expression was determined using Cufflinks v. 2.0.2. software. Differentially expressed genes were identified as described in [1].

### 2.4 Identification of the affected pathways from the differentially expressed genes

Online tool DAVID (http://david.abcc.ncifcrf.gov/) [3], [4] was used to find out the affected pathways among the differentially expressed gene lists. The gene lists were uploaded with selecting the background as all the genes of *E. coli*. Functional Annotation Chart was visualized using the p-value threshold of 0.01 and a minimum number of genes of 4.The information regarding the affected pathways was obtained from Kyoto Encyclopedia of Genes and Genomes (KEGG) within the analysis in DAVID, using the mentioned thresholds.

## Figures and Tables

**Table 1:** Pathways most affected upon Ni stress.

Term	%	*p*-value
**Down-regulated genes**		
Ribosome	9	5.9 x 10^−19^
Purine metabolism	8.5	1.3 x 10^−14^
Flagellar assembly	5.5	3.2 x 10^−9^
Sulfur metabolism	3.5	1.4 x 10^−8^
Pyrimidine metabolism	5	6 x 10^−8^
ABC transporters	7	9.1 x 10^−6^
Biosynthesis of siderophore groups non ribosomal peptides	2	6.1 x 10^−5^

**Up-regulated genes**		
Two component system	5.5	3.4 x 10^−7^
Phenylalanine metabolism	2.7	4.3 x 10^−5^

Pathway analysis has been performed from the list of differentially expressed genes using the online tool DAVID and as per information from KEGG. The threshold was settled to ≥ 4 genes being involved in a given pathway.

% : involved genes/total of up- or down-regulated genes.

*p*-value : modified Fisher exact *p*-value.
