# Supplementary material for: Data set for transcriptome analysis of Escherichia coli exposed to nickel
Source: Data Brief. 2016 Sep 7;9:314–7. doi: 10.1016/j.dib.2016.08.069 (PMC5026706; doi:10.1016/j.dib.2016.08.069)
Supplement: Supplementary file 1 — Supplementary material [file mmc1.docx]

Agnès Rodrigue

Associate Professor, HDR, INSA Lyon

Team Leader “Membrane Trafficking and Signaling in Bacteria”

Microbiology Adaptation and Pathogenicity Unit

Bat. Lwoff – 10 rue Dubois

69622 Villeurbanne cedex FRANCE

[Agnes.rodrigue@insa-lyon.fr](mailto:Agnes.rodrigue@insa-lyon.fr)

Tél. : +33 4 72 44 79 80

To the Editor

*Data in Brief*

Villeurbanne, 21^st^ July 2016

Dear Editor,

This is an original paper which has neither previously, nor simultaneously, in whole or in part, been submitted anywhere else.

We confirm that all of the reported work is original, all authors have seen and approved the final version submitted, all prevailing local, national and international regulations and conventions, and normal scientific ethical practices, have been respected, and consent is given for publication in Data in Brief, if accepted.

With regards,

Agnès Rodrigue

Reviewers' comments:

Reviewer #1: I recommend revisions to the format and content of the manuscript to conform to the Data in Brief requirements. Please see comments and track changes in 'DIB-D-16-00531 Edited.docx' attachment (available for you to download and update via the View Reviewer Attachments link). Note that the cross-check (similarity) result was 20% - please ensure that text and data files are not duplicated (cite related article instead).

We revised the manuscript in accordance with the reviewer’s comments. We made changes in order to conform to the journal editorial policy where it was mentioned in the edited manuscript. We removed interpretations and conclusive remarks. We hope the new version fulfills the requirements.

-the abstract has been changed

-the “Data” section has been shortened

-the “Experimental Design, Materials and Methods” part has been revised in order to avoid redundancy with the related research article.

We removed any duplicated text. We ensure that the data files, Table and Figure are original and were not published elsewhere.
